# Young women neurosurgeons and challenges – need for equity and diversity

**DOI:** 10.3389/fsurg.2022.889375

**Published:** 2022-07-27

**Authors:** Adriana Rodrigues Libório dos Santos, Ana Cristina Veiga Silva, Joaquim Fechine de Alencar Neto

**Affiliations:** ^1^ Neurosurgery Department of Ipanema Federal Hospital, Rio de Janeiro-RJ, Brazil; ^2^ Neuroscience Post-Graduate Program, Federal University of Pernambuco, Recife, Pernambuco, Brazil; ^3^ Faculty of Medical Sciences, Unifacisa University Center, Campina Grande, Brazil

**Keywords:** young women, neurosurgery, gender inequality, motherhood, career

## Introduction

We live in a patriarchal social system, where men hold power and dominate in leadership positions, moral authority in family domain, in politics, control of goods and properties and consequent social privilege. It's no different in the academic environment, much less in the neurosurgical specialty (1).

If not, let us look at the historical abnegation of the role played by pioneering women in the neurosurgery field, as Spetzler pointed out on his paper (1), which became a landmark in the discussion of gender disparity in neurosurgery, on an historical redemption attempted to rescue and recognize “the founding mothers” of the specialty.

Neurosurgery is a specialty with a long training and learning journey, with postgraduate specializations, fellowships, congresses, continuous updating courses and academic actions. It is recognized that there must be total dedication and genuine passion for the specialty to achieve job satisfaction as a neurosurgeon. The young neurosurgeons, at the beginning of their career, face several obstacles. However, when training is adequate, they become technically capable of performing highly complex surgical procedures with good results for patients.

## Gender disparity and challenges for young neurosurgeons

Young neurosurgeons still face multiples challenges despite insertion in competitive and high differentiate labor market. Challenges that can be intensified if we consider gender disparity since neurosurgery is one of the least equitable and slowest increase of women practicing surgery (2).

The structural male chauvinism faced by residents and physicians is notorious and getting more apparent when analyzing the reduced rate of representation which the female figure occupied in leadership roles or prominence in surgical conferences (3). Thus, especially in surgical areas, having as exponent neurosurgery, despite the increasing number of female residents who aim to follow this career, the gender disparity between still significant, as Brazilian medical demography demonstrated women representing only about 8.6% of neurological surgeon's experts in 2018 (4).

Women are underrepresented in leadership positions and must carry out leadership training and always prove their skills to be equated with colleagues (5).

Women neurosurgeons suffer all kinds of judgments, pressure and teasing in their workplace, from superiors and colleagues, differently from their male counterparts, which gives rise to gender disparity. The discrimination becomes even more evident when the pregnancy and maternity issues interfere with the productivity of young female residents or neurosurgeons, revealing an unsupportive work environment and even hostile behavior from the surgical department members (6).

Lifestyle is a major concern for both sexes, as the specialty requires a lot of study and training time, dedication, abnegation, and physical burden (1, 7), within this routine, motherhood is not considered compatible with a successful career in neurosurgery, however with organization it is possible.

## Discussion

One of great difficulties that widens this disparity is challenging conciliation between family and career planning in medical training. If a neurosurgeon become pregnant during the period of residence, the maternity leave, and childcare stress added to an unsupportive practice environment often led to discrimination and questions about technical capabilities and skills. Among the obstacles that can be listed for this condition advancing in the neurosurgery career, less opportunities for leadership positions and, above all, little time for care and breastfeeding (6), since breaks and adequate places for lactating residents are not provided for in the services.

In addition, another point of great relevance that should be reported is the discrepancy between races when evaluating the characteristics of neurosurgery residents. Analyzing physicians of the neurosurgery residency program in the US, for twelve years, Maqsood and collaborators (8) found that the largest contingent was white and caucasians. This difference points out the maintenance of structural racism and specialty elitization that, by associating itself with lack of representativity and social inequity, makes it almost impossible to others ethnic groups the interest in neurosurgery and the chance to build a solid career in the profession. The situation of black women in this situation is even more worrying (9, 10). Thus, the need for institutions develops mechanisms that provide an equitable relationship between physicians and residents, especially neurosurgery, is an urgent point, making the work of these vulnerable groups more dignified and, above all, demystifying prejudices rooted in our society.

Increasing representation, responsibility, and valorization of women in neurosurgery are highly notorious and necessary, although their contributions are not fully known, they have played an important role, in breaking “glass ceilings” and diversifying the workforce, especially in the recruitment of distinguished students, inspired by their trajectories, almost always, of resilience, determination, and perseverance (11).

As one of the important mentors in neurosurgery field, Robert Spetzler mentioned that “gender is less important than the general fact that we are all neurosurgeons” (1). Mentoring is important and having a distinguished leader who encourages you in various stages of training, in surgical and academic settings is essential so that there is no gender disparity in neurosurgery services.
